# Cities as an Enabler for Strengthening Cancer Care

**DOI:** 10.1200/GO.20.00607

**Published:** 2021-06-15

**Authors:** Susan Henshall, Raul Doria, Cary Adams, Sanchia Aranda

**Affiliations:** ^1^ City Cancer Challenge, Geneva, Switzerland; ^2^ Centro Medico La Costa, Asunción, Paraguay; ^3^ Union for International Cancer Control, Geneva, Switzerland; ^4^ School of Health Sciences, University of Melbourne, Melbourne, Australia; ^5^ City Cancer Challenge, Geneva, Switzerland

For most countries, the pace of change for cancer is too slow to achieve Sustainable Development Goal 3.4 (to reduce premature mortality from noncommunicable diseases [NCDs] by one third).^1^ The most recent review of progress found that trends in the risk of death from 2010 to 2016 for liver cancer, colorectal cancer, and the residual group of other cancers in women and men, and lung cancer in women indicated that < 10% of all countries were on track for a one-third reduction.^2^

Despite many low- and middle-income countries (LMICs) advancing in the development of national cancer control plans, governments are often constrained in their capacity to deliver on the full spectrum of cancer services with most plans unfunded. A study of publicly available cancer-related plans from 158 countries found that a mere 45% (70 of 155) of countries have specified radiotherapy services in their plans.^3^ This figure decreases to 30% (7 of 23) of cancer-related plans in low-income countries. Similarly, guidelines for cancer treatment and reference to the WHO List of Essential Medicines are specified in the cancer-related plans of 53% (82 of 155) and 30% (45 of 150) of countries, respectively.^3^ Beyond service delivery, other health system building blocks such as health workforce, financing, and governance issues remain poorly addressed. Consequently, cancer care is often fragmented and difficult to navigate.

In response, the Union for International Cancer Control (UICC), with its network of more than 1,100 member organizations, established a new program of action to mobilize and engage civil society in driving access to cancer treatment.^4^ This built on UICC's advocacy efforts to advance global resolutions for palliative care and an expanded essential medicines list, adopted by the WHO Member States in 2014 and 2016, respectively, as well as the overarching Cancer Resolution adopted in 2017.

First launched in 2017, the city-based initiative City Cancer Challenge leveraged the reputation of UICC as a trusted civil society partner with the power to convene diverse stakeholders. Through its extensive global membership in LMICs, relationships with patient groups, national cancer institutes, cancer registries, professional associations, and industry partners, UICC was able to rapidly engage a first set of so-called key learning cities. Specifically, Asuncion, Paraguay; Cali, Colombia; Kumasi, Ghana; and Yangon, Myanmar, were selected against three measures of readiness assessed using data collected by WHO, other UN Agencies, and UICC members reporting on cancer and NCD control.^5-9^ These measures were evidence of readiness to support core cancer services, evidence of an active civil society and engagement with other sectors, and evidence of progress in the implementation of national commitments to cancer and NCD control measures.

The convening of the major decision makers in the cancer care ecosystem in these cities provided the initial impetus for creating a locally led, multistakeholder, and multilevel governance approach to assess local needs and design data-driven solutions to cancer care. With City Cancer Challenge Foundation (C/Can) established as a standalone foundation in 2019, and now operational in nine cities (Table 1), it is timely to assess the learnings to date.

**TABLE 1:**
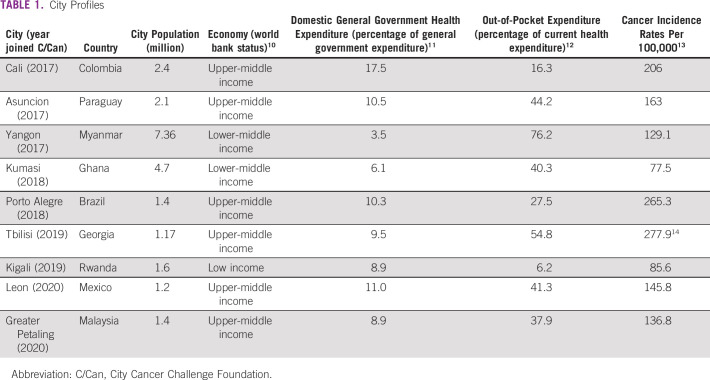
City Profiles

International health organizations have most often worked directly via the national ministry of health as the central actor in the governance, delivery, and financing of health care at various levels (central, regional, and local). The recognition of the role of cities in achieving the Sustainable Development Goals through goal 11 has provided an alternative framework whereby global, national, and local health goals are aligned and cities are vehicles of implementation. In this way, cities' proximity to local users and providers is best used to drive locally led solutions that are context-specific. City-led initiatives for hypertension, tobacco control, and diabetes,^15-17^ as well as other examples of NCD prevention initiatives from the WHO Healthy Cities innovation platform,^18^ are using this approach to tackle NCDs at a local level. In each case, intersectoral action, political commitment, multilevel governance, and community engagement have been identified as key enablers to address NCD risk factors, their social determinants, and related inequities.^18^ C/Can's early experience in cities validates these findings in the cancer care context.

As the complexity of health systems grows, and with the increasing presence of private oncology providers, intersectoral partnerships and facilitated stakeholder communications can promote the elimination of cancer-related inequities.^18,19^ Georgia is a key example wherein the current health care system is a blend of privately owned hospitals and state-owned small-scale health care providers.^20^ Taking a multisectoral approach to address fragmentation in care and improve the quality, standardization and financial coverage of cancer care were identified by local stakeholders as major priorities in Tbilisi through the city-wide needs assessment, which involved 174 professionals from 27 institutions including the Ministry of Refugees and Accommodation of Georgia, Tbilisi City Hall, the National Center for Disease Control and Public Health of Georgia, and the Georgian Patients Union. An agreed objective was to increase accessibility to internationally recognized treatment standards for oncologic patients and, specifically, to reduce out-of-pocket spending on cancer services. The response was that in 2020, the Government of Georgia announced the expansion of cancer medicines covered by the Universal Healthcare Program together with an increase in financial coverage per patient, per annum.^21^ The approach in Georgia underscores how coordinated engagement and collaboration across sectors can help to address collective priorities and, more broadly, in this case, to support the Government of Georgia's commitment to provide universal health coverage.^20,22^ Moreover, it supports the adoption of a multilevel governance approach (local, subnational, and national actors) to promote dialogue and inform priority setting. This approach can also be a catalyst for filling city-level gaps and securing investment in public health services, by communicating data-driven priorities to regional and national governments. This was the case in Colombia, whereby the city-wide needs assessment involving 186 health professionals in Cali highlighted gaps in access to cancer services in the public system and health system fragmentation.^23^ In alignment with the national strategy^24^ and international best practices,^25^ this prompted a feasibility study for the implementation of a Comprehensive Cancer Care Center within the reference tertiary public hospital, which services the entire Valle del Cauca region. As a result, Colombia's Ministry of Health and the regional government of Valle del Cauca have jointly supported the creation of the center through investment in infrastructure and equipment for medical imaging, radiotherapy, and pathology to strengthen existing services and meet future demand.^26,27^

Maintaining high-level commitment, even during changes in political leadership, is central to sustainable system improvements as well as financing and delivery of outcomes. In Paraguay, a change in President in 2018 risked deprioritizing cancer in the national health agenda and withdrawal of support for C/Can's activities in Asuncion. Through strong leadership from the bipartisan City Executive Committee (convened by C/Can) and targeted communications, the first national cancer law was adopted under the new government's leadership.^28,29^ Even with this support, however, ongoing threats to implementation must be managed. For example, the strains placed on the national health system and local health professionals by the global coronavirus pandemic require new strategies to support local leaders to sustain activities.

Finally, community partnerships are critical in developing solutions that respond to the needs of the most vulnerable. Local governments and providers have everyday contact with health care professionals and patients with cancer and their families and are closest to their concerns and priorities. Across C/Can's first seven cities, more than 900 patients and 1,200 health care professionals have been engaged in the city needs assessment process. Additionally, local professional associations, patient associations, and civil society representatives are engaged from the outset as part of the local decision-making structure.

All C/Can cities have demonstrated readiness and commitment to adopt these enablers through a memorandum of understanding between government authorities, civil society, and C/Can. This agreement also commits cities to lead on collaborative, evidence-informed planning and execution of city-level projects that will drive common goals to improve cancer outcomes. In the case of Yangon, by the end of 2020, resource-appropriate breast and cervical cancer guidelines and multidisciplinary team methodologies in pathology, pain management, and radiotherapy have been developed through the C/Can process with city and national stakeholders and international experts. These collaborative efforts also formed the basis for a new resource, which will be scaled to all cities. The guide for developing resource-appropriate breast cancer management guidelines, developed in partnership with the Breast Health Global Initiative,^30^ responds to the urgent need to reduce inequities in access to breast cancer diagnosis, multimodality treatment, and palliative care services. The development of these guidelines provides a roadmap to improve the coordination of cancer care and supports cities to lead on gender-responsive policies that meet the specific needs and barriers to care faced by women in their communities. Importantly, these local efforts can be scaled through initiatives such as the WHO Global Breast Cancer Initiative and Global Strategy to Accelerate the Elimination of Cervical Cancer.^31,32^

Informed by these insights garnered in the first cities, this special series presents the C/Can approach as it currently stands following 4 years of iterative and adaptive change. The first paper in the series provides a detailed assessment of this framework, specifying the activities at each stage, as well as the outputs, processes, and practices across the lifecycle of the intervention.^29^ Specific attention is given to the actions taken to activate, strengthen, and multiply effective health services, workforce, and infrastructure. Three papers in this series address pathology, radiotherapy, and multidisciplinary care and collectively show how C/Can applies a multistakeholder and multilevel governance approach to respond to cities' needs. Case studies will also be used to showcase knowledge exchange between individuals, institutions, and cities on planning and uptake of best practices and highlight common challenges to successful implementation.

In the final paper, C/Can's fit-for-purpose Monitoring, Evaluation, and Learning Framework to measure progress and impact over time will be described. Integral to an ongoing process of continuous learning and iteration, the framework is designed to provide data-driven insights into what is working well, as well as what adjustments may be needed to drive improvements in the approach.

As C/Can continues to validate this city-led approach, further refinement will be required. Technical cooperation to strengthen each of the six pillars of the health system is at the core of C/Can's model. As C/Can grows its reach, the unique needs of each new city require recalibrating the delicate balance between increasing efficiency by replicating existing city solutions and further customization of technical cooperation offerings for local context. Second is a further understanding of the complex adaptive nature of health systems. In the current context with all cities managing the additional strain of the global pandemic, C/Can will need to go beyond the six pillars and focus on their interconnectedness and the political, social, and economic influences affecting health system resilience in each city. Third is the need to build data capacity at the individual, institutional, and policy levels. Quality data are a prerequisite to be responsive to the local context and leverage the expertise, resources, and commitments of C/Can's network stakeholders and partners. Experience of running data collection processes in seven of the nine cities has highlighted the challenges of collecting quality systems-level data. These include low levels of data literacy, lack of organizational processes for the ethical use and sharing of data, and policy barriers to effective and responsible use of systems data. As an emerging area of work, in 2021, C/Can is piloting new data quality processes and policies as well as a customized data platform. A collaboration with the University of Malaya, Greater Petaling, will explore how to support data-and-analytics activities at scale in C/Can cities in ways that build local data capacity and inform policy change.

C/Can will continue to integrate on-the-ground learnings to scale, adapt, and validate its approach, and contribute to a robust evidence base for the effective implementation of locally adapted cancer care solutions in LMICs.
